# Isolation and Characterization of an Aluminum-resistant Mutant in Rice

**DOI:** 10.1186/s12284-016-0132-3

**Published:** 2016-11-11

**Authors:** Shuo Liu, Huiling Gao, Xiaoyan Wu, Qiu Fang, Lan Chen, Fang-Jie Zhao, Chao-Feng Huang

**Affiliations:** State Key Laboratory of Crop Genetics and Germplasm Enhancement, College of Resources and Environmental Science, Nanjing Agricultural University, Nanjing, 210095 China

**Keywords:** Aluminum toxicity, Aluminum resistance, Mapping, Mutant, Rice, Screening

## Abstract

**Background:**

Aluminum (Al) toxicity represents a major constraint for crop production on acid soils. Rice is a high Al-resistant plant species among small-grain cereals, but its molecular mechanisms of Al resistance are not fully understood. We adopted a forward genetic screen strategy to uncover the Al-resistance mechanisms in rice. In this study, we screened an ethylmethylsulfone (EMS)-mutagenized library to isolate and characterize mutants with altered sensitivity to Al in rice.

**Results:**

Treatment of an Al-intolerant indica variety Kasalath with 20 μM Al induced root swelling. This phenotype could be suppressed by the addition of aminoethoxyvinylglycine (AVG, an ethylene synthesis inhibitor), suggesting that increased production of ethylene is responsible for the root swelling under Al stress. By utilizing the root swelling as an indicator, we developed a highly effective method to screen Al-sensitive or -resistant mutants in rice. Through screening of ~5000 M2 lines, we identified 10 Al-sensitive mutants and one Al-resistant mutant *ral1* (*r*esistance to *al*uminum 1). *ral1* mutant showed short root phenotype under normal growth condition, which was attributed to reduced cell elongation in the mutant. A dose-response experiment revealed that *ral1* mutant was more resistant to Al than wild-type (WT) at all Al concentrations tested. The mutant was also more resistant to Al when grown in an acid soil. The mutant accumulated much lower Al in the root tips (0–1 cm) than WT. The mutant contained less Al in the cell wall of root tips than WT, whereas Al concentration in the cell sap was similar between WT and the mutant. In addition to Al, the mutant was also more resistant to Cd than WT. Quantitative RT-PCR analysis showed that the expression levels of known Al-resistance genes were not increased in the mutant compared to WT. Genetic analysis indicated that the Al-resistance phenotype in *ral1* mutant was controlled by a single recessive gene mapped on the long arm of chromosome 6.

**Conclusions:**

We have developed a highly efficient method for the screening of rice mutants with altered Al sensitivity. We identified a novel mutant *ral1* resistant to Al by this screening. The increased resistance of *ral1* to Al toxicity is caused by the reduced Al binding to the cell wall of root tips and the responsible gene is mapped on the long arm of chromosome 6.

## Background

Aluminum (Al) is the third most abundant element after oxygen and silicon in the soil and comprises about 7 % of the earth’s crust. Most Al exists as insoluble aluminosilicates or oxides, which are non-toxic to plants grown in mildly acidic or neutral soils. However, in acid soils with a pH of 5.5 or lower, Al is solubilized and released into the soil as Al^3+^ which will inhibit plant growth. As a consequence, Al toxicity is one of the most severe global problems of acid soils especially since these soils comprise approximately 30 % of the world’s arable land (von Uexkull and Mutert 1995). To cope with Al toxicity on acid soils, some plant species have evolved Al-resistance mechanisms.

Numerous studies indicate that organic acid anions play critical roles in the detoxification of Al (Ma 2000; Ryan et al. 2001; Ma et al. 2001). In response to Al, plants secrete organic acids such as malate, citrate and oxalate to chelate Al and thereby alleviate Al toxicity. A number of plant species are documented to secrete different organic acids to detoxify Al. For instance, wheat (*Triticum aestivum*), *Arabidopsis thaliana* and oilseed rape (*Brassica napus*) release malate for Al detoxification (Delhaize et al. 1993; Ligaba et al. 2006; Hoekenga et al. 2003), whereas snapbean (*Phaseolus vulgaris*), rice bean (*Vigna umbellata*), maize (*Zea mays*), and soybean (*Glycine max*) secrete citrate to detoxify Al (Miyasaka et al. 1991; Pellet et al. 1995; Yang et al. 2000; Liu et al. 2013; Yang et al. 2006). Oxalate is exuded from roots of buckwheat, tomato and spinach (*Spinacia oleracea*) under Al stress (Ma et al. 1997; Zheng et al. 1998; Yang et al. 2005; Yang et al. 2011). Recently, genes responsible for the Al-activated secretion of malate and citrate have been identified in plants (Furukawa et al. 2007; Magalhaes et al. 2007; Sasaki et al. 2004), but genes required for oxalate release are still unknown.

In addition to organic acid-based mechanisms of Al detoxification, recent molecular genetic studies on the model plants rice and Arabidopsis have revealed some novel Al-resistance mechanisms. Through a forward genetic screen, two research groups identified C2H2-type zinc-finger transcription factors STOP1 and ART1 involved in Al resistance in *Arabidopsis thaliana* and rice, respectively (Yamaji et al. 2009; Iuchi et al. 2007). STOP1/ART1 regulates the downstream Al-resistance genes to confer Al resistance. *STAR1* and *STAR2/ALS3* that encode a nucleotide-binding domain and a transmembrane domain of an ABC (ATP-binding cassette) transporter, respectively, interact with each other to form a complex and then transport UDP-glucose for the cell wall modification to detoxify Al (Larsen et al. 2005; Huang et al. 2009a; Huang et al. 2010). *ALS1* encoding a half-size ABC transporter is involved in the sequestration of Al into the vacuoles (Huang et al. 2012a; Larsen et al. 2007), which suggests that in addition to Al accumulator species, normal plant species also possess internal Al detoxification mechanisms. By examining the function of the downstream genes of ART1, Ma’s group also identified several additional Al-resistance genes involved in various processes of Al detoxification in rice (Xia et al. 2010; Chen et al. 2012; Yokosho et al. 2011; Xia et al. 2013). More recently, Arenhart et al. (2013) reported that a transcription factor ASR5 (abscisic acid, stress and ripening) is required for Al detoxification in rice. They further demonstrated that ASR5 regulates Al resistance through direct binding to the promoters of target genes including the key Al-resistance gene *STAR1* (Arenhart et al. 2013; Arenhart et al. 2014).

Japonica rice is the most Al-resistant plant species among small-grain cereal crops. Compared to japonica cultivars, indica cultivars are less resistant to Al. A number of quantitative trait loci (QTLs) responsible for the differential Al resistance between japonica and indica varieties have been identified (Xue et al. 2007; Nguyen et al. 2003; Ma et al. 2002; Nguyen et al. 2002; Nguyen et al. 2001; Wu et al. 2000). However, due to the minor effect of each QTL on the Al resistance, cloning of the responsible genes by a map-based cloning approach is greatly hampered. Alternatively, mutant screening followed by map-based cloning of the responsible gene is an effective strategy to identify new genes and discover novel mechanisms in plant species with known genome sequence including rice. Through this strategy, several rice mutants with increased sensitivity to Al were isolated and the responsible genes were cloned and characterized (Yamaji et al. 2009; Ma et al. 2005; Huang et al. 2009b; Huang et al. 2009a; Huang et al. 2012b). Previously, the method used to screen Al-sensitive mutants was based on the measurement of root length of each plant before and after Al treatment. This screening method is time-consuming and labor-intensive (Ma et al. 2005). In this study, we developed an easy and efficient screening method for isolation of Al-sensitive or Al-resistant mutants to further examine the Al-resistance mechanisms in rice. We chose an indica variety for mutagenesis and mutant screening because this variety showed less resistant to Al and Al-induced root swelling phenotype, which allows us to screen both Al-sensitive and Al-resistant mutants efficiently. Through this screen, we identified a rice mutant with increased resistance to Al toxicity. The mutant was characterized physiologically and genetically.

## Results

### Development of an Efficient Screening Method for Isolation of Rice Mutants with Altered Al Sensitivity

**Fig. 1:**
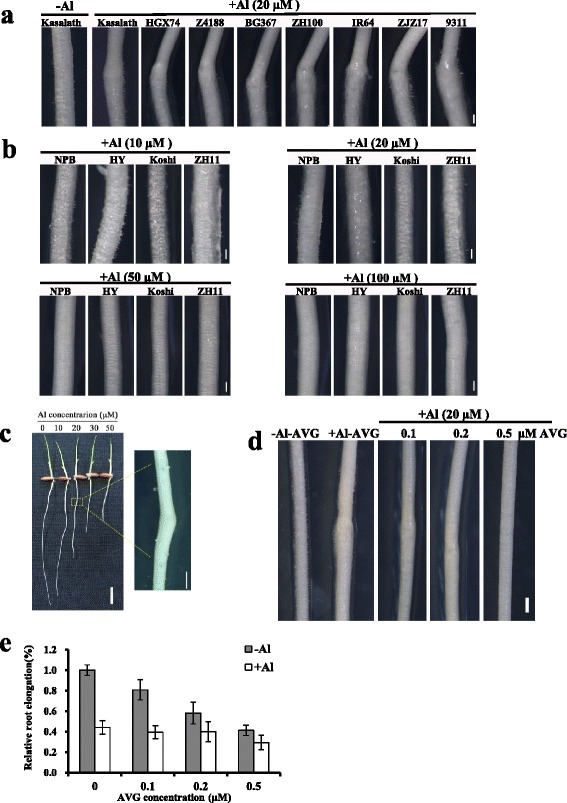
Effect of Al and aminoethoxyvinylglycine (AVG) on root swelling and root elongation. **a** Root swelling phenotype of indica cultivars under Al stress. Roots were exposed to –Al or + Al (20 μM) condition for 24 h. Scale bar = 500 μm. **b** Al stress did not induce root swelling in japonica cultivars. Roots were exposed to a series of Al concentrations (10, 20, 50 or 100 μM) for 24 h. Scale bar = 500 μm. **c** Root swelling occurrence of Kasalath cultivar under different Al concentrations. Roots were exposed to a series of Al concentrations (0, 10, 20, 30, or 50 μM) for 24 h. Scale bar in left and right panel is 1 cm and 500 μm, respectively. **d**, **e** Effect of AVG on Al-induced root swelling (**d**) and root elongation (**e**). Roots were treated with 0, 0.1, 0.2 or 0.5 μM AVG in combination with 0 or 20 μM Al for 24 h. Scale bar = 500 μm

Using the laborious root length method, Ma et al. (2005) and Huang et al. (2009b) screened less than 2000 rice lines, which was far from saturation, implying that more Al-sensitive or Al-resitant mutants await to be screened. We found that Al treatment was able to induce root swelling in all 8 Al-intolerant indica cultivars including Kasalath used in this study (Fig. 1a), which could be used as a marker to indicate the root length before Al treatment. Unlike the indica cultivars, Al-resistant japonica cultivars did not show the Al-induced root swelling phenotype at all Al concentrations tested (Fig. 1b). These results demonstrated that Al toxicity can induce root swelling in Al-intolerant cultivars (indica cultivars), but not in Al-resistant cultivars (japonica cultivars). A dose-response experiment revealed that the optimal Al concentration for the induction of root swelling in the Kasalath cultivar is 20 μM Al (Fig. 1c), which inhibited root elongation of Kasalath by 54 %. Increased biosynthesis of the plant hormone ethylene under Al stress is suggested to be responsible for the Al-induced root swelling phenotype in soybean (Kopittke et al. 2015). To investigate whether the Al-induced root swelling in the rice cultivar Kasalath was caused by the ethylene, we used an ethylene synthesis inhibitor, aminoethoxyvinylglycine (AVG), to reduce the ethylene production in Al-treated roots. Results showed that the addition of AVG could attenuate the root swelling induced by Al toxicity, and increase of AVG concentration to 0.5 μM was able to fully suppress the root swelling phenotype (Fig. 1d). Although AVG treatment alone inhibited root elongation in a concentration-dependent manner, supply of both Al and AVG did not show additive effect on the inhibition of root elongation, suggesting that AVG could ameliorate the Al toxicity to some extent (Fig. 1e). Together, these results suggest that Al-induced root swelling in Kasalath cultivar might be caused by increased ethylene production.

The root swelling marker allowed us to compare the root elongation difference after exposure to Al through visual inspection without the need to measure the root length. We generated a mutant library with Kasalath genetic background through ethylmethylsulfone (EMS) mutagenesis, and screened mutants with altered Al sensitivity by treating M_2_ seedlings with 20 μM Al for 3 days. Root elongation from the swelling marker was assessed visually. Seedlings with root elongation either less than half or more than two folds of the normal length were selected as Al-sensitive or Al-resistant candidate mutants, respectively. We also retained mutants without the occurrence of root swelling after exposure to Al. By this method, we easily screened ~5000 M2 lines and obtained 243 candidate mutants. In the second screening, M_3_ generation of each candidate mutant was evaluated for their resistance to Al based on relative root elongation (RRE). As a result, 10 Al-sensitive and one Al-resistant mutants were confirmed. In the present study, the Al-resistant mutant *ral1* (*r*esistance to* al*uminum 1) was selected for further characterization.

### Response of *ral1* Mutant to Al and Other Metals

**Fig. 2:**
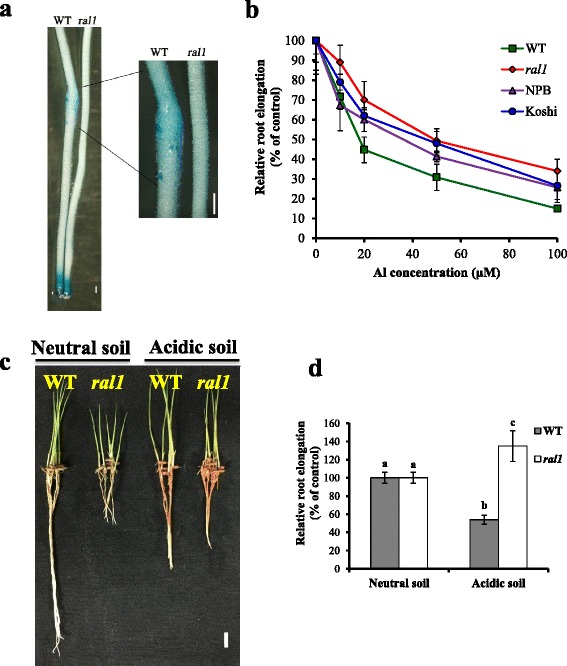
Comparison of Al resistance phenotype between WT and *ral1* mutant. **a** Evans blue staining. Roots of WT and the mutant were exposed to 20 μM Al for 24 h and then stained with 0.025 % Evans blue. Scale bar = 500 μm. **b** A dose-response experiment of Kasalath (WT), *ral1* mutant, NPB and Koshi. Seedlings were exposed to a 0.5 mM CaCl_2_ solution (pH 4.5) containing 0, 10, 20, 50 or 100 μM Al for 48 h. Relative root elongation (RRE) was used to evaluate their resistance to Al. Data are means ± SD (*n* = 7). **c**, **d** Comparison of root growth between WT and the mutant on different soils. Germinated seeds were grown on neutral soil (pH 6.6) or acidic soil (pH 4.1) for 6 days. Scale bar = 1 cm. Data are means ± SD (*n* = 6). Means with different letters are significantly different (*P* < 0.05, Tukey test)

The *ral1* mutant did not exhibit the typical root swelling phenotype in response to Al treatment (Fig. 2a). Evans blue staining showed that Al treatment induced more cell death in WT than in the mutant (Fig. 2a). This result was consistent with the notion that *ral1* mutant was more resistant to Al than WT.

**Fig. 3:**
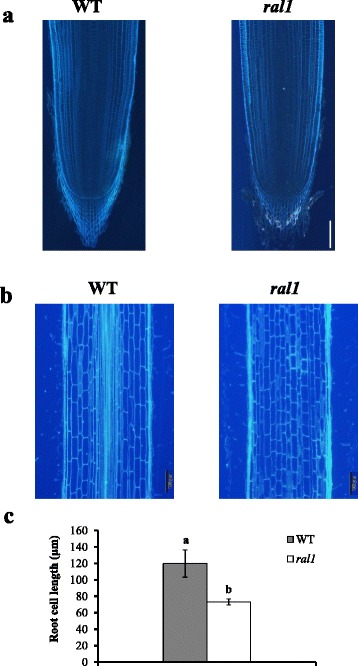
Comparison of root meristem and cell length between WT and the mutant. **a**, **b** Root meristem (**a**) and mature zones (2 cm from tips) (**b**) of WT and the mutant were longitudinally excised and compared. **c** Comparison of cell length of WT and the mutant in the mature zones. Cell length of exodermal cells was calculated and compared. Data are means ± SD (*n* = 6). Means with different letters are significantly different (*P* < 0.05, Student’s t-test)

To further compare the Al resistance phenotype of *ral1* mutant with WT, we exposed roots of WT and the mutant to a series of Al concentrations. Root elongation of the mutant was slower than that of the wild-type in the absence of Al (WT, 40 ± 6.8 mm/48 h vs *ral1*, 25 ± 3.7 mm/48 h). Longitudinal sections of root meristem and mature zones showed that while root meristem morphology of the mutant did not differ from that of WT (Fig. 3a), root cell length in the mature zone was shorter in the mutant than in WT (Fig. 3b and c), indicating that short root phenotype in the mutant was mainly caused by the defective cell elongation. However, in the presence of 10, 20, 50 and 100 μM Al, root elongation of WT plants was respectively inhibited by 28, 55, 69 and 85 %, whereas that of the mutant was inhibited by 11, 30, 51 and 66 %, respectively (Fig. 2b). This result indicates that *ral1* mutant was more resistant to Al than WT. We also compared the Al resistance of *ral1* mutant with two Al-resistant japonica cultivars NPB and Koshi, and the results showed that the mutant was slightly more resistant to Al than the Al-resistant cultivars (Fig. 2b). Since the differential Al resistance between japonica and indica varieties is controlled by multiple QTLs (Ma and Furukawa 2003), it is unlikely that the high Al resistance in *ral1* mutant was caused by the mutation of one of the Al-sensitive QTLs. To further confirm the increased resistance of *ral1* mutant to Al, we grew the mutant and WT plants on an acid soil with a pH of 4.1 and a neutral soil with a pH of 6.6. In the neutral soil, the growth rate of the mutant was lower than that of WT (Fig. 2c). In the acid soil, the root growth of WT plants was inhibited by 54 %, whereas that of the mutant was stimulated in comparison with its growth on the neutral soil (Fig. 2d).

**Fig. 4:**
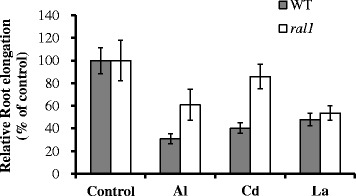
Sensitivity to other metals. Seedlings of WT and *ral1* mutant were exposed for a 0.5 mM CaCl_2_ solution (pH 4.5) containing 0, 20 μM Al, 5 μM Cd, 5 μM La for 48 h. Data are means ± SD (*n* = 8). Relative root elongation (RRE) was used to evaluate their sensitivity to Al. Means with different letters are significantly different (*P* < 0.05, Tukey test)

To examine whether the increased resistance of *ral1* mutant to Al is specific, we exposed roots of WT and the mutant to other toxic metals including Cd and La. At 5 μM La, inhibition of root elongation in the mutant was not significantly different from that in WT (Fig. 4). By contrast, 5 μM Cd inhibited 60 and 14 % of the root elongation in WT and the mutant, respectively. These results revealed that the mutant was also more resistant to Cd.

### Al Accumulation Pattern of *ral1* Mutant

**Fig. 5:**
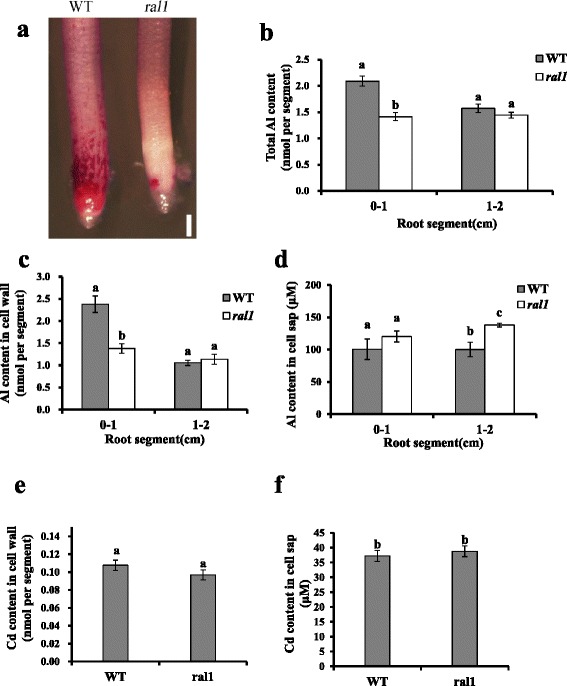
Comparison of Al and Cd accumulation pattern between WT and *ral1* mutant. **a** Eriochrome Cyanine R staining of WT and *ral1* mutant after 20 μM Al treatment for 24 h. Scale bar = 200 μm. **b**-**d** Comparison of total Al content (**b**), Al content in cell wall (**c**), and Al content in cell sap (**d**) in different root segments of WT and the mutant. Seedlings were exposed to a 0.5 mM CaCl_2_ solution containing 20 μM Al at pH 4.5 for 6 h. Root tips (0–1 cm) and basal roots (1–2 cm) were excised for Al determination. **e**, **f** Comparison of Cd content in cell wall (**e**) and Cd content in cell sap (**f**) in root tips of WT and the mutant. Seedlings were exposed to a 0.5 mM CaCl_2_ solution containing 5 μM Al at pH 4.5 for 6 h. Data are means ± SD (*n* = 3). Means with different letters are significantly different (*P* < 0.05, Tukey test)

To investigate whether Al accumulation in *ral1* mutant was altered or not, roots of WT and the mutant were exposed to 20 μM Al for 24 h, and then the roots were stained by an Al indicator Eriochrome Cyanine R (ER). While the root tip including root cap, meristem and transition zones was heavily stained in the wild-type, the mutant displayed much lighter color in the root tip (Fig. 5a), indicating that *ral1* mutant accumulated less Al in root tips than WT. Al inhibits root elongation within hours (Ryan et al. 1993; Sivaguru et al. 1999). To determine whether WT and the mutant differ in Al accumulation in a short term, we exposed the roots to Al for 6 h and then measured the total Al content in root tips (0–1 cm) and basal roots (1–2 cm). Results showed that while total Al content in basal roots of *ral1* was similar to that of WT, the mutant accumulated significantly less Al in root tips than WT (Fig. 5b). These results suggest that reduced Al accumulation in the root tips was the likely cause of increased Al resistance in the mutant.

We further fractionated the root segments into cell wall and cell sap to investigate which component accumulated less Al in the mutant. Results showed that Al content in the cell wall of the root tips was much lower in *ral1* than in the wild type (Fig. 5c), whereas Al accumulation in the cell sap was similar between WT and the mutant (Fig. 5d). In the basal roots, Al accumulation in the cell wall of the mutant did not differ from that of the WT, although the Al content in the cell sap was slightly increased in the mutant compared to the WT (Fig. 5c and d). These results suggest that the cell wall of *ral1* mutant root tips had a reduced capability to bind Al, resulting in increased resistance to Al.

Since *ral1* mutant was more resistant to Cd as well, we want to know whether Cd accumulation pattern in the mutant was altered or not. Measurement of Cd content in the cell wall and cell sap revealed that the Cd accumulation in the two components of root tips was not significantly different between WT and the mutant (Fig. 5e and f), which suggested that the increased resistance of the mutant to Cd and Al might be through different mechanisms.

### Effect of *RAL1* Mutation on The Expression of Al-resistance Genes in Rice

**Fig. 6:**
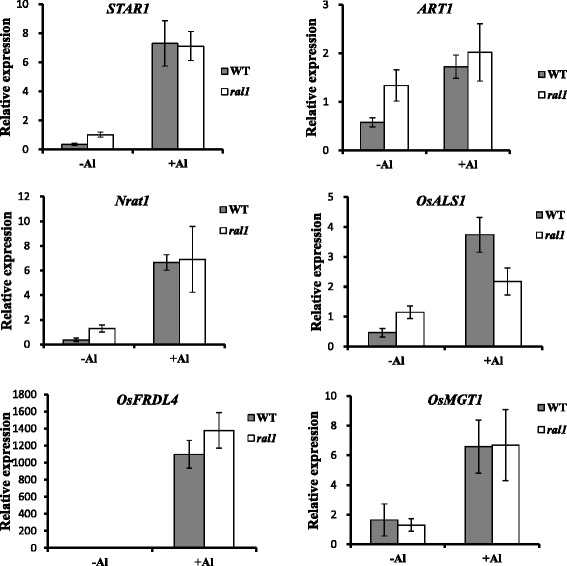
Expression pattern of Al-resistance genes in WT and *ral1* mutant. Seedlings were exposed to 0 or 20 μM Al for 6 h and root tips (0-2 cm) were excised for RNA isolation and cDNA synthesis. The expression of Al-resistance genes indicated on the top of each panel was determined by real-time RT-PCR and *Histone H3* was used as an internal control. The data were normalized to the expression of each gene in the mutant without Al treatment

To investigate whether the *ral1* mutant had altered expression of Al-resistance genes, roots of WT and the mutant were exposed to 0 or 20 μM Al for 6 h and the expression of Al-resistance genes was determined. Although the expression levels of *STAR1*, *ART1*, *Nrat1* and *ALS1* genes in the mutant were higher than those in WT without Al treatment (Fig. 6), these genes were expressed at a similar level in WT and the mutant under Al stress. The expression of the other Al-resistance genes *OsFRDL4* and *OsMGT1* did not differ between WT and the mutant under either –Al or + Al conditions (Fig. 6). These results suggest that increased Al resistance of *ral1* was not through elevated expression of Al-resistance genes.

### Genetic Analysis of *ral1* Mutant and Molecular Mapping of *RAL1* Gene in Rice

Genetics analysis of *ral1* mutant was performed by using an F2 population from a backcross between *ral1* and its wild type Kasalath. Of 148 F2 seedlings, 43 seedlings showed Al resistance (RRE > 65 %) and short root phenotype in the absence of Al (Fig. 6a), whereas the remaining 105 seedlings with normal root elongation were intolerant to Al (RRE ≤ 65 %). The segregation pattern was consistent with 1:3 ratio (χ^2^ = 0.62, *P* > 0.05), suggesting that the Al resistance and short root phenotype in *ral1* mutant was controlled by a single recessive gene.

**Fig. 7:**
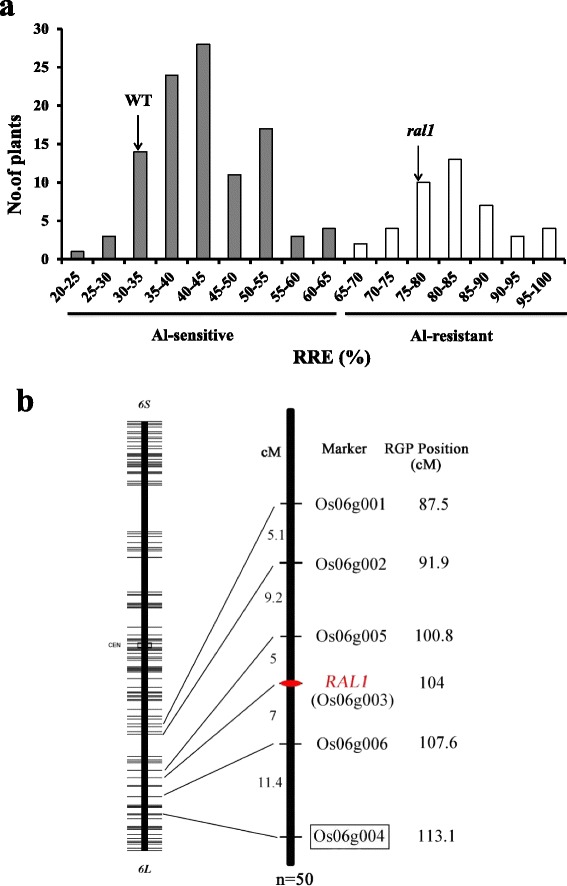
Genetic analysis and molecular mapping of *ral1*. **a** Frequency distribution of Al sensitivity and short root phenotype in *ral1*/WT F2 population. A total 148 F2 seedlings were exposed to –Al condition for 24 h and exposed to + Al (20 μM) condition for a further 24 h. Normal-root plants (Gray bar) and short-root plants (White bar) was determined based on that root elongation was more than 13 mm and less than 10 mm at the first 24 h, respectively. Relative root elongation (RRE) was used to evaluate their sensitivity to Al. **b** Molecular mapping of *ral1*. Bulked segregant analysis was used to determine the chromosome location of *ral1* and a polymorphic marker Os06g004 boxed was found to be linked. Fifty F_2_ plants with short roots and five additional markers were used to further map the gene. Left bar represents the chromosome 6 downloaded from RGP website (http://rgp.dna.affrc.go.jp). Genetic distance (cM) between neighboring makers and RGP genetic position (cM) were also indicated

To map the responsible gene, we constructed an F2 population derived from a cross between the mutant and an indica cultivar HJX74. Since the Al resistance and short root phenotype was co-segregated and controlled by the same gene (Fig. 7a), we evaluated the phenotype of each F2 plant based on the short root appearance. In the *ral1*/HJX74 F2 population, 64 of 247 plants showed the short root phenotype, which also agreed to a single gene segregation pattern (χ^2^ = 0.03, *P* > 0.05). Bulked segregant analysis with 54 polymorphic markers covering the whole rice genome was used to determine the chromosome location of *RAL1* and a SSR polymorphic marker Os06g004 on the long arm of chromosome 6 was found to be linked to the gene (Fig. 6b). To further map the gene, 50 F2 mutants and six polymorphic markers around *RAL1* gene were used. Linkage analysis indicated that *RAL1* gene was located between the two markers Os06g005 and Os06g006 on the long arm of chromosome 6, with a genetic distance of 5.0 and 7.0 cM, respectively (Fig. 6b). The marker Os06g003 on 104 cM position of RGP map was tightly linked to the gene.

## Discussion

In this study, we found that Al toxicity can induce root swelling in Al-intolerant indica cultivars including Kasalath (Fig. 1a), but not in Al-resistant japonica cultivars (Fig. 1b). Al-induced root swelling can also occur in soybean (Kopittke et al. 2015), which is less resistant to Al than rice. These observations suggest that Al-induced root swelling is not a universal phenomenon, but appears to occur preferentially in Al-intolerant plant species. In support of this hypothesis, Al-induced root swelling does not occur in the Al-resistant mutant *ral1* isolated in the present study (Fig. 2a). The burst of ethylene evolution induced by Al stress is suggested to be responsible for the Al-induced root swelling in soybean (Kopittke et al. 2015). Our results also showed that inhibition of ethylene production by AVG treatment could suppress the Al-induced root swelling and ameliorate Al-induced root growth inhibition (Fig. 1d and e). These suggest that Al-induced ethylene production that causes the root swelling is conserved in Al-intolerant varieties of both dicots and monocots.

Because of the difference among individual plants in the seed germination and root elongation rate, it is difficult to directly compare the root elongation rate between plants after Al treatment. Therefore, root length of individual plants is usually measured before and after Al treatment for the calculation of root elongation during the treatment period (Ma et al. 2005; Huang et al. 2009b). This screening method is therefore labor-intensive and inefficient. In the present study, we found that Al induced root swelling in the indica variety Kasalath. The point of swelling serves as a marker for the initial position of root growth before Al treatment. Root elongation from this marker after Al treatment could be assessed quickly by visual inspection without the need to measure the root length twice. By this simple and efficient method, we are able to screen ~5000 M2 lines and isolated 11 mutants with altered Al sensitivity. Among them, one had a mutation in the coding sequence of *OsALS1*, whilst the others did not have mutations in the known Al-resistance genes (Data not shown), which suggests that our new screening method is feasible and highly efficient.

Although we evaluated Al resistance based on relative root elongation and root growth of *ral1* mutant was slower than that of WT under control condition, we do not think that less root growth without Al treatment is necessarily correlated with higher relative root elongation under Al. In fact, we also isolated several additional short root mutants with altered Al sensitivity. Although they showed slower root growth under control condition, these mutants are more sensitive to Al than WT instead of more resistance to Al because they had lower relative root elongation under Al treatment (Data not shown). Therefore, higher relative root elongation in *ral1* mutant compared to WT under both hydroponic culture and acidic soil growth conditions indicates that *ral1* is an Al-resistant mutant. Further work revealed that *ral1* accumulated less Al in the cell wall of root tips than in WT, while the Al content in the cell sap was similar between the mutant and WT. This difference suggests that reduced Al binding to the cell wall is responsible for the increased Al resistance in the mutant. Al-resistant cultivars of maize, rice and common bean are also documented to accumulate less Al in the cell wall of root tips than the Al-sensitive ones (Eticha et al. 2005; Yang et al. 2008; Rangel et al. 2009), and differences in the proportion of low-methylated pectin are suggested to be responsible for the varietal differences in Al resistance. It remains to be demonstrated whether the increased resistance of *ral1* mutant to Al is also through the alteration of cell-wall pectin content and/or the degree of pectin methylation. Recently, Zhu et al. (2012) reported that mutation of *XTH31*, a xyloglucan endotransglucosylase-hydrolase gene, also results in increased resistance to Al in *Arabidopsis thaliana*, which is caused by a decrease of root xyloglucan content and associated cell wall Al accumulation in the mutant. Unlike *xth31* mutant where the Al content of the whole roots is decreased nearly three folds, our *ral1* mutant had reduced Al accumulation only in the root tips. In addition, *ral1* mutant is resistant not only to Al but also to Cd. These data suggest that Al-resistance mechanism of *ral1* is likely to be different from that of *xth31*.

Although organic acid anions do not play a major role in the detoxification of Al in rice (Ma et al. 2002), Al-induced citrate secretion through OsFRDL4 transporter contributes to the Al resistance (Yokosho et al. 2011). Our expression analysis showed that the expression of *OsFRDL4* in *ral1* mutant was similar to that in WT (Fig. 5), suggesting that increased resistance of the mutant to Al was not through Al-activated citrate exudation pathway. Expression analysis of other known Al-resistance genes in rice also reveals that the increased resistance of *ral1* to Al does not appear to be through enhanced expression of those Al-resistance genes. Subsequently, we used a map-based cloning approach to isolate the responsible gene and mapped the gene to the long arm of chromosome 6. Although the Al-resistance gene *STAR1* is also located on the long arm of chromosome 6 (Ma et al. 2005; Huang et al. 2009a), the genetic distance of *RAL1* and *STAR1* in RGP genetic map (http://rgp.dna.affrc.go.jp) was different. *RAL1* was mapped between Os06g005 (100.8 cM) and Os06g006 (107.6 cM) (Fig. 7b), while *STAR1* gene is located at 115.6 cM. Furthermore, the coding sequence and expression level of *STAR1* was not altered in the mutant (Fig. 5 and data not shown). These indicate that *RAL1* is a novel Al-resistance gene in rice.

## Conclusion

We develop a highly efficient method to screen Al-sensitive or -resistant mutants in rice. Using this method, we identify a novel mutant resistant to Al. Physiological analysis reveals that decreased Al binding capacity to the cell wall is responsible for the increased resistance of *ral1* mutant to Al. *RAL1* gene is mapped on the long arm of chromosome 6, and cloning of the gene is currently undertaken.

## Methods

### Al-induced Root Swelling in Different Rice Cultivars

For root swelling experiment, 8 indica and 4 japonica cultivars were used. The indica varieties were Kasalath, Huagengxian74 (HJX74), Zhong4188 (Z4188), BG367, Zihui100 (ZH100), IR64, Zhongjiazai17 (ZJZ17) and 9311, and the japonica cultivars were Nipponbare (NPB), Huayang (HY), Koshihikari (Koshi) and Zhonghua11 (ZH11). Five-day old seedlings were exposed to a 0.5 mM CaCl_2_ solution containing 0, 10, 20, 50 or 100 μM AlCl_3_ for 72 h. Root swelling phenotype was observed under a stereo microscope (SZX7, Olympus).

### Construction of a Mutant Library and Screening of Rice Mutants with Altered Al Sensitivity

For mutant library construction, seeds of an Al-intolerant indica cultivar (cv. Kasalath) were soaked in tap water for 8 h at room temperature. After soaking, the seeds were treated with 1 % ethylmethylsulfone (EMS) solution for 8 h, which inhibited seed germination rate by 50–60 %. The seeds were then washed to remove residual EMS and incubated at 37 °C overnight. Germinated seeds were sowed and grown in a paddy field to harvest M_2_ seeds from each M_1_ plant. For initial mutant screening, 16 seeds each of 36 M_2_ line were put in 96-well PCR plates that were floated on a 0.5 mM CaCl_2_ solution in a 10 l container at 25 °C. After growth for 5 days, seedlings were exposed to a 0.5 mM CaCl_2_ solution (pH 4.5) containing 20 μM AlCl_3_ for 3 d. Because 20 μM AlCl_3_ could induce swelling of each root, root elongation after Al treatment was roughly estimated based on the distance between the root swelling position and root tips by eye. Root elongation with decreased or increased more than 2 folds was used as a parameter to select Al-sensitive or Al-resistant candidate mutants, respectively. Mutants that did not show root swelling after exposure to Al were also retained.

For the second screening, M_3_ generation of candidate mutants were harvested and used to evaluate their sensitivity to Al. Briefly, M_3_ seeds of each candidate mutant were soaked in deionized water for 2 d at 37 °C. The seeds were then placed on a net floating on a 0.5 mM CaCl_2_ solution in a 2.5 l container. After growth at 25 °C for 3 d, 10 seedlings each were exposed to a 0.5 mM CaCl_2_ solution containing 0, 20 or 50 μM AlCl_3_ at pH 4.5 for 48 h. The root length was measured with a ruler before and after Al treatment. Relative root elongation (RRE) was expressed as (root elongation with Al treatment/root elongation without Al) × 100. Lines with lower or higher RRE than WT at either 20 or 50 μM AlCl_3_ were regarded as Al-sensitive or -resistant mutants, respectively.

### Aminoethoxyvinylglycine (AVG) Treatment

For aminoethoxyvinylglycine treatment, Kasalath seedlings were exposed to a 0.5 mM CaCl_2_ solution containing 0, 0.1, 0.2 or 0.5 μM AVG and with or without 20 μM AlCl_3_ for 24 h. Root swelling and Al resistance between different treatments were compared.

### Root Meristem and Cell Length Comparison

To compare the difference of root meristem and cell length between WT and *ral1* mutant, root tips and basal roots (2 cm from tips) were excised and immersed in FAA solution (70 %) under vacuum condition for 12 h. The roots were treated with 70 % ethanol and then subjected to an acetone series (70, 80, 90, 95 and 100 %), and thereafter transferred to a series of acetone/Epon812 resin solution with different ratios (3:1, 1:1 and 1:3). Finally, the roots were embedded in Epon812 resin for at least 48 h and cut longitudinally into 5 μm slices with an ultramicrotome (Lecia EM UC7). Cell wall autofluorescence and cell length of root sections was observed with a microscope (BX 53 microscope, Olympus).

### Evaluation of Resistance to Al and Other Metals

For a dose–response experiment, 8 seedlings each of WT (cv. Kasalath) and *ral1* mutant were exposed to a 0.5 mM CaCl_2_ solution containing 0, 10, 20, 50 or 100 μM Al Cl_3_ at pH 4.5 for 48 h. Relative root elongation (RRE) described above was used to evaluate the resistance to Al. For comparison of the resistance of WT and the mutant to other metals, seedlings were exposed to 0.5 mM CaCl_2_ solutions containing 0, 20 μM AlCl_3_, 5 μM CdCl_2_ or 5 μM LaCl_3_ for 48 h and RRE was used to evaluate their resistance to the metals.

To further evaluate the Al resistance, germinated seeds of WT and *ral1* mutant were sown in an acidic soil (pH 4.1) and a neutral soil (pH 6.6), which were collected from different regions of China. After growth for 6 days, the root length was measured and compared.

### Evans Blue and Eriochrome Cyanine R Staining

Five-day-old seedlings of WT and *ral1* mutant were exposed to a 0.5 mM CaCl_2_ solution (pH 4.5) containing 20 μM Al for 24 h, and then the roots were stained with 0.025 % Evans blue or 0.1 % Eriochrome Cyanine R for 10 min. After staining, roots were washed with distilled water for 10 min and photographed by the stereo microscope.

### Determination of Al and Cd Content

To determine Al content in roots, seedlings of WT and *ral1* mutant were exposed to a 0.5 mM CaCl_2_ solution containing 20 μM Al for 6 h. Root tips (0–1 cm) and basal roots (1–2 cm) were excised and placed in a plastic tube containing 0.5 ml of 2 M HNO_3_. The tubes were votexed five times during a 48 h period. The Al concentration in the solution was measured by inductively coupled plasma mass spectrometry (ICP-MS, Perkin Elmer NexIon300). Measurement of the Al or Cd content in different compartments was performed according to Xia et al. (2010). Briefly, root tips or basal roots of WT and the mutant treated with 20 μM Al or 5 μM Cd for 6 h were put in Milipore Ultrafree-MC Centrifugal filter units. The samples were first centrifuged at 3000 g for 10 min at 4 °C to remove the apoplastic fluid and then the rest samples were kept at −80 °C overnight. The frozen samples were thawed at room temperature and centrifuged at 15,000 rpm for 10 min to collect the cell sap. The obtained cell sap was transferred to a new 1.5 ml tube containing 0.5 ml of 0.1 M HNO_3_ for the Al determination. The residual cell wall was washed with 70 % ethanol, vortexed and centrifuged at 15,000 rpm for 5 min. After three rounds of washing, the cell wall was immersed in 0.5 ml of 2 M HNO_3_ for at least 24 h with occasional votexing. The Al or Cd concentration in the solution was determined by ICP-MS.

### RNA Isolation and Quantitative RT-PCR Analysis

To examine the expression pattern of Al-resistance genes in WT and *ral1* mutant, 5-day-old seedlings were exposed to a 0.5 mM CaCl_2_ solution containing 0 or 20 μM Al at pH 4.5 for 6 h and then root tips (0–2 cm) were excised for RNA isolation. Total RNA was extracted using a Universal Plant Total RNA Extraction Kit (BioTeke, http://www.bioteke.com). One microgram of total RNA was used for first strand cDNA synthesis by using HiScript II Q Select RT SuperMix (Vazyme, http://www.vazyme.com), following the instruction manual of the kit with an oligo(dT)_20_ primer. The primer sequences for real-time RT-PCR analysis of Al-resistance genes were as follows: *STAR1* (Forward, 5′-TCGCATTGGCTCGCA CCCT-3′; Reverse, 5′-TCGTCTTCTTCAGCCGCACGAT-3′), *ART1* (Forward, 5′-CAGTGCTTCTCGTGGGTCTT-3′; Reverse, 5′-CCTGTGCGTGAAGAACCAC T-3′), *Nrat1* (Forward, 5′-GCAAAGCACCACTATCAG-3′; Reverse, 5′-GAA CTTGAGTAGAGGGATG-3′), *OsALS1* (Forward, 5′-GGTCGTCAGTCTCTGCC TTGTC-3′; Reverse, 5′-CCTCCCCATCATTTTCATTTGT-3′), *OsFRDL4* (Forward, 5′-CGTCATCAGCACCATCCACAG-3′; Reverse, 5′-TCATTTGCGAA GAAACTTCCACG-3′), *OsMGT1* (Forward, 5′-GAGGGTGGAGTTTGGGAA GC-3′; Reverse, 5′-CCCTGGAGCCTGACGACGATG-3′), *OsCDT3* (Forward, 5′-ATGTACAACCCTCCGGCGGC-3′; Reverse, 5′-TCAGCAGCAGCAGAGGC ATTCG-3′), and the sequences of internal control *Histone H3* were 5′-GGTCAACTTGTTGATTCCCCTCT-3′ and 5′-AACCGCAAAATCCAAAG AACG-3′. Data were collected in accordance with CFX96 Touch real-time PCR detection system (BioRad).

### Genetic Analysis and Molecular Mapping

For genetic analysis of *ral1* mutant, an F2 population from a cross between mutant and WT (cv. Kasalath) was used. Seedlings of F2 and their parents were exposed to a 0.5 mM CaCl_2_ solution at pH 4.5 for 24 h. Root length was measured before and after treatment, and calculated root elongation was used to evaluate the short root phenotype of each individual in comparison with their parents. The seedlings were then exposed to a 0.5 mM CaCl_2_ solution (pH 4.5) containing 20 μM Al for another 24 h. Relative root elongation (RRE) was used to determine the sensitivity of each seedling to Al stress.

For mapping of *ral1* mutant gene, an F2 population from a cross between *ral1* and another indica cultivar HJX74 was used. Bulked segregant analysis (BSA) by pooling equal amounts of DNA from 20 short-root or 20 normal-root plants was used to determine the chromosome location of the mutant gene. The polymorphic markers used for BSA were derived from published high-density SSR markers (McCouch et al. 2002). To further map the gene, linkage analysis was carried out by using 50 F2 mutants and six polymorphic markers. The primer sequences of the six markers are as follows: Os06g001 (Forward, 5′-ACAAGCAAAGCAAGTCCATTC-3′; Reverse, 5′-TGGTGAGAACTCCCAAGGCT-3′), Os06g002 (Forward, 5′-GAATTGCCGTA TGTCGGAGTC-3′; Reverse, 5′-GGACAAATAATGGGAGCCTTG-3′), Os06g003 (Forward, 5′-AATCTTCATGCCTTTGTCGC-3′; Reverse, 5′-GGTGCTATGTTG GTTTTCCTG-3′), Os06g004 (Forward, 5′-GGTAAATGGACAATCCTATGGC-3′; Reverse, 5′-GGTAAATGGACAATCCTATGGC-3′), Os06g005 (Forward, 5′-GGCATCCAATTTTACCCCTC-3′; Reverse, 5′-AAATGGAGCATGGAGGTC AC-3′), and Os06g006 (Forward, 5′-GATGATCCATGCTTTGGCC-3′; Reverse, 5′-TTCCAGCAGAAAGAA GACGC-3′). Multipoint linkage analysis was performed using MAPMAKER Version 3.0 (Lander et al. 2009).

## Abbreviations

ABC: ATP-binding cassette
Al: Aluminum
AVG: Aminoethoxyvinylglycine
BSA: Bulked segregant analysis
EMS: Ethylmethylsulfone
ER: Eriochrome Cyanine R
ICP-MS: Inductively coupled plasma mass spectrometry
*ral1*: Resistance to aluminum toxicity
RRE: Relative root elongation
WT: Wild-type
